# Editorial: Species diversity in evolution and replacement of teeth informing clinical therapies

**DOI:** 10.3389/fdmed.2023.1225665

**Published:** 2023-06-21

**Authors:** Christopher A. McCulloch

**Affiliations:** Faculty of Dentistry, University of Toronto, Toronto, ON, Canada

**Keywords:** regeneration, periodontal, tooth morphology, mineralization, junctional epithelium

**Editorial on the Research Topic** Species diversity in evolution and replacement of teeth informing clinical therapies

Three articles in this Research Topic of Frontiers in Dental Medicine consider how knowledge of species diversity from an evolutionary standpoint may be used to broaden and deepen current and future approaches for natural tooth replacement and for periodontal tissue regeneration in humans. Insights obtained from the signaling systems that govern tooth formation could, as suggested by Popowics and Mulimani of the University of Washington (Seattle), provide new approaches for tooth development and replacement. They address long-standing questions in dental development and evolution in the context of how phylogenetic signals and mechanical function impact morphological variations of teeth.

Conceivably, obtaining a deeper understanding of the regulatory mechanisms that maintain the dental lamina for lifelong tooth generation in animals with multiple sets of teeth in succession (polyphyodonts) could suggest new approaches for promoting regeneration of teeth beyond the diphyodont dentition of humans. For pursuing this approach in a practical sense, much more work and in-depth insights will be needed to identify what the repertoires of signals arising from the dental lamina that enable *de novo* tooth formation. Further, from an evolutionary perspective, the authors consider that functional adaptations with respect to phylogenetic signals in primitive mammals, impact teeth development within Carnivora, the Superfamily Suoidea and Primates. The authors also point out that autologous stem cells may be able to promote the formation of dental tissues, which has been previously considered and is very much work in progress. But Popowics and Mulimani now point out that evolutionarily conserved developmental pathways may be combined with more advanced research methods (e.g., iPSCs and organoids), which may in turn suggest new methods for whole tooth regeneration, and possibly with well-defined coronal and root structural features.

Theodoro et al. from Odontologia de Araçatuba, UNESP, Brazil and her colleagues examine some of the fundamental biology underlying how the junctional epithelium maintains periodontal health and they also take into account evolutionary processes that may be important for the development of this structure and possibly, for developing new treatment approaches. Notably, teeth in mammals penetrate protective epithelial layers. In the dentogingival junction, tight adhesion of the marginal gingiva to the root surface is crucial for periodontal health. Subgingival sites that are colonized by destructive microbial species often exhibit destruction of the underlying connective tissues, in part because of the failure of the marginal gingival sealing system, which is seen in periodontitis.

Theodoro et al. consider how oral epithelium, sulcular epithelium, and junctional epithelium have evolved to maintain the adhesion of the dentogingival junction as a defense system against periodontal diseases. They focus on the expression of certain junctional epithelial adhesion molecules and enamel proteins that normally provide tissue adhesion to roots and that guide enamel development, and that are disturbed in periodontitis. Some of the molecular adhesion systems that they describe include Lam332/SCPPPQ1. But they also consider that certain proteins involved in hydroxyapatite mineralization such as amelotin and ODAM may anchor multiprotein adhesion complexes to mineralized tooth surfaces. The ability of these proteins to maintain soft tissue attachment to enamel or root surfaces in spite of inflammation, indicates that these proteins may prevent breakdown of the dento-gingival junction. This research focus may provide exciting possibilities for regeneration and repair of the dento-gingival junction. Degradation of these proteins likely enhances the progression of periodontitis and developing methods to prevent their breakdown and possibly to enhance their abundance in healing periodontal wounds may suggest important advances in periodontal therapy.

**Figure 1:**
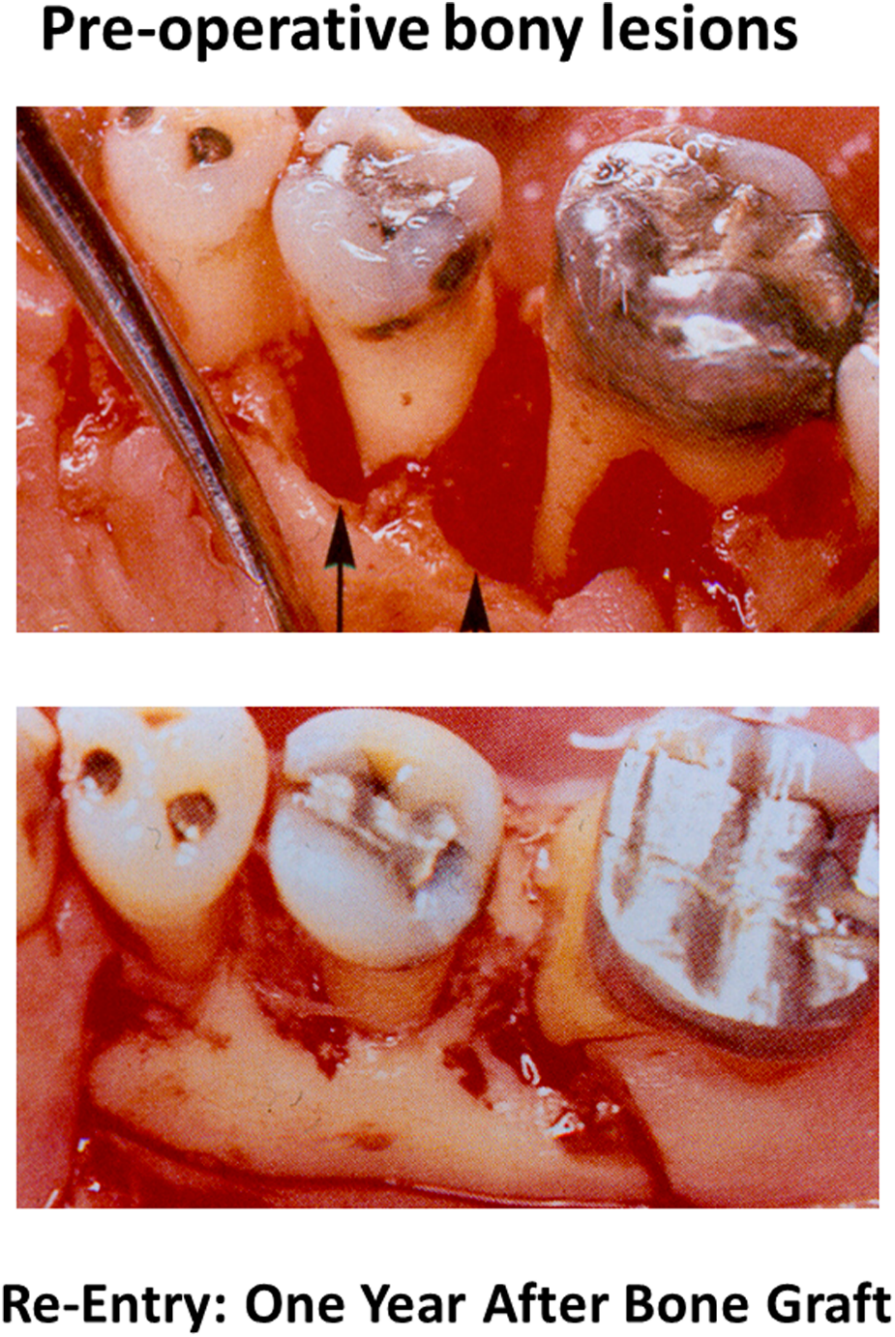
Clinical photographs of infrabony periodontal lesions prior to treatment with autogenous bone graft (top) and re-exposure of the lesions one year later (bottom) showing apparent new bone formation.

In the third article, Sone and McCulloch of the University of Toronto focusses on how the tightly regulated process of mineralization of collagen fibrils in the periodontal ligament, may be critical for enabling regeneration of fibrillar collagen attachments to root surfaces that have been affected by periodontitis. Current clinical approaches for treatment of periodontitis are limited by the ability to restore the insertion of periodontal ligament fibers into mineralized tissue attached to cementum or dentine. An example of this limitation is shown in the accompanying figure. Even when bone formation is optimized following use of periodontal surgical regenerative procedures, there are still major shortcomings on how much new attachment can be achieved (Figure 1). An important limitation in current surgical methods is the near impossibility of regenerating cementum.

In this context, much of Sone and McCulloch's current research is focused on the study of the sharply delineated interface of mineralization at the junctions of periodontal ligament fibers with cementum. The regulatory mechanisms that preserve the structure and function of these junctions are not understood, but clearly, there is a high level of spatio-temporal precision in their assembly. From the standpoint of clinical dentistry, the fibrillar collagen/mineralization interface will directly impact future approaches to periodontal regeneration and orthodontic treatment. Sone and McCulloch consider why the treatment outcomes seen in experimental periodontitis as seen in various animal models are so much less impressive than observations in human studies and brings into this discussion how species differences in the structure of mineralized tissue/fibrillar insertions may affect these outcomes. They also raise the long-standing knowledge gap about the lineage structure of the progenitor cells in periodontal regeneration and whether there are specific and possibly well-differentiated cell populations that are needed in relatively high numbers to enable restoration of collagen/mineralization interfaces.

Finally, they consider whether certain soluble molecules, many of which are already identified (e.g., bone sialoprotein), and other extracellular matrix and mechanical signals, may orchestrate the formation of this critical interface. Analogous to the conclusions in tooth development and junctional epithelium that are mentioned in the two previous articles, Sone and McCulloch note that for effective design of new regenerative methods and materials, it will be important that we bring together evolutionary-based observations of periodontal tissue structures along with in-depth understanding of how myriad signaling events control gene expression in order to enable worthwhile improvements in periodontal regeneration.

Taken together, these three articles indicate that there is good reason to believe that advances in the biological underpinnings of tooth morphology, junctional epithelium and control of mineralization, could advance the technological developments needed for regeneration of oral tissues. Real progress will likely rely on bringing these fundamental insights into the clinical arena and assessing how far these ideas can advance the field.

